# Diverting tyrosine: Data from untargeted metabolic analysis of tomato fruit accumulating L-DOPA

**DOI:** 10.1016/j.dib.2020.106678

**Published:** 2020-12-19

**Authors:** Dario Breitel, Paul Brett, Saleh Alseekh, Alisdair R. Fernie, Eugenio Butelli, Cathie Martin

**Affiliations:** a Department of Metabolic Biology and Biological Chemistry, The John Innes Centre, Norwich Research Park, Norwich NR4 7UH, UK; b Max-Planck-Institut fur Molekulare Pflanzenphysiologie, Am Muhlenberg 1, 14476 Potsdam-Golm, Germany

**Keywords:** Tomato, Metabolic analysis, L-DOPA, Tyrosine, GC-MS, LC-MS

## Abstract

L-DOPA, also known as Levodopa or L-3,4-dihydroxyphenylalanine, is synthesised in plants from the amino acid tyrosine, through oxidation. Conversion of tyrosine to L-DOPA constitues the first step of betalain biosynthesis in plants. Recently, the gene responsible for this step was identified in beetroot, *BvCYP76AD6*, that is the source of yellow and purple betalain pigments. Overexpression of this gene, specifically in tomato fruit, led to accumulation of L-DOPA that otherwise is not detectable [1]. Co-expression of the Arabidopsis transcription factor, *AtMYB12*, in fruit, increased L-DOPA levels further. To study the metabolic changes in these fruit, we performed untargeted metabolite analysis of ripe fruit: GC-MS was performed to identify changes in primary metabolites, LC-MS analysis was used to identify alterations in specialised metabolites. These data can be used to study the impact of diversion of tyrosine in fruit, accompanied by the accumulation of L-DOPA *in planta* and to identify new biological roles associated with the accumulation of these metabolites.

## Specifications Table

**Table:** 

Subject	Omics: Metabolomics
Specific subject area	Metabolic analysis of tomato fruit accumulating L-DOPA
Type of data	Table
How data were acquired	Metabolite analyses of tomato fruit accumulating L-DOPA was carried out by GC-MS and UPLC-MS. For the GC-MS analysis we used of a DB-35 column and Leco Pegasus HT TOF-MS. Chromatograms were analysed by using Chroma TOF 4.5 (Leco) and TagFinder 4.2 software. For the UPLC-MS analysis we used an HSS T3 C18 reversed phase column (100 × 2.1 mm i.d., 1.8-µm particle size; Waters), and a Q-Exactive Orbitrap mass detector. Molecular masses, retention times, and associated peak intensities were extracted from the raw files using RefinerMS (version 5.3; GeneData), and Xcalibur software (Thermo Fisher Scientific).
Data format	Raw
Parameters for data collection	Fruit was harvested 7 days post breaker. For GC-MS analysis mass spectra were recorded at 20 scans s^−1^ with m/z 70-600 scanning range. For LC analysis, the spectra were recorded using full scan mode with negative ion detection, covering a mass range from m/z 100 to 1500. The resolution was set to 25,000, and the maximum scan time was set to 250 ms.
Description of data collection	For GC-MS analysis, chromatograms and mass spectra were evaluated by using Chroma TOF 4.5 (Leco) and TagFinder 4.2 software. For LC-MS analysis, molecular masses, retention times, and associated peak intensities were extracted from the raw files using RefinerMS (version 5.3; GeneData), and Xcalibur software (Thermo Fisher Scientific)
Data source location	Max-Planck-Institut fur Molekulare Pflanzenphysiologie Potsdam-Golm Germany Samples grown and collected at: John Innes Centre Norwich United Kingdom
Data accessibility	With the article
Related research article	[1]

## Value of the Data

• These data are useful as defining total metabolite changes in fruit engineered to divert tyrosine into alternative products
• People investigating the control of metabolic flux in ripening tomato fruit will find these data interesting.
• These data could be useful for researchers investigating the central role of tyrosine and regulation of tyrosine levels in plants on both primary and secondary metabolism.

## 1 Data Description

To investigate the metabolic effect of accumulation of L-DOPA in tomato, GC-MS (Table 1) and LC-MS (Table 2) analyses were undertaken on fruit, seven days post breaker. An untargeted approach was adopted to identify as many compounds as possible.

For GC-MS analysis, three lines of tomato producing L-DOPA (CYP76AD6) were analysed- CYP76AD6#1, with 3 independent, biological replicates (a-c), and CYP76AD6#2 and CYP76AD6#3, with four independent, biological replicates each (a-d). In addition, fruit from lines crossed with plants overexpressing *AtMYB12* (CYP76AD6XMYB12) and accumulating higher levels of L-DOPA were analysed [1]. CYP76AD6#1XMYB12 consisted of four independent, biological replicates (a-d), and CYP76AD6#2XMYB12 consisted three independent, biological replicates (a-c). As controls, four independent, biological replicates of wild type (wt) fruit seven days post-breaker were analysed (a-d), and three biological repeats of *AtMYB12*- overexpressing fruit (MYB12-a, MYB12-b and MYB12-c). The GC-MS analysis identified predominantly primary metabolites and a total of 77 compound-associated m/z signals were quantified.

For the LC-MS analysis, three lines of tomato producing L-DOPA were analysed- CYP76AD6#1, CYP76AD6#2, and CYP76AD6#3, with five independent, biological replicates each (a-e). In addition, fruit were sampled from lines crossed with plants overexpressing *AtMYB12*. Six independent, biological replicates of CYP76AD6#1XMYB12 fruit were analysed (a-f) and three independent biological replicates from CYP76AD6#2XMYB12 plants (a-c). As controls, five independent, biological replicates of wild type (wt) fruit were analysed (a-e), together with five independent, biological replicates of *AtMYB12*- overexpressing fruit (MYB12-a-e). The LC-MS analysis identified mostly specialised, secondary metabolites and a total of 91 compound-associated m/z signals were extracted.

## 2 Experimental Design, Materials and Methods

### 2.1 Plant material

Plant material was obtained as described in [1]. Briefly, transgenic tomato plants (cv. Money Maker), overexpressing the beetroot *BvCYP76AD6* coding DNA sequence (CDS; CYP76AD6), under the control of the fruit-specific E8 promoter were grown in greenhouses at the John Innes Centre (UK). In addition, *BvCYP76AD6*-overexpressing lines, were crossed with plants overexpressing *AtMYB12* [2] to generate F1 plants from the different parental lines (CYP76AD6XMYB12). Fruit were harvested seven days post breaker. Placenta and seeds were removed, prior to being immersed in liquid nitrogen and stored at -80°C.

Samples were ground in liquid nitrogen, freeze-dried overnight and extracted (30mg ml^−1^) in 80% methanol with ribitol 1.5mg l^−1^. Samples were shaken at room temperature for 30 min, followed by 10 min sonication and 10 min centrifugation in 4°C.

### 2.2 GC-MS analysis

The ribitol-methanol fruit extract was derivatised for 90 min at 37°C (in 50 µl of 20 mg ml^−1^ methoxyamine hydrochloride in pyridine) followed by a 30 min treatment at 37°C with 120 µl of MSTFA (*N*-Methyl-*N*-(trimethylsilyl)trifluoroacetamide, *N*-Trimethylsilyl-*N*-methyl trifluoroacetamide). The GC-MS system used included a gas chromatograph coupled to a time-of-flight mass spectrometer (Leco Pegasus HT TOF-MS). For sample injection, a Gerstel Multi Purpose autosampler was used. Helium was used as carrier gas at a constant flow rate of 2 ml s^−1^ and gas chromatography was performed on a 30 m DB-35 column. The injection temperature was 230°C and the transfer line and ion source were set to 250°C. The initial temperature of the oven (85°C) increased at a rate of 15°C min^−1^ up to a final temperature of 360°C. After a solvent delay of 180 sec, mass spectra were recorded at 20 scans s^−1^ with m/z 70-600 scanning range. Chromatograms and mass spectra were evaluated by using Chroma TOF 4.5 (Leco) and TagFinder 4.2 software.

### 2.3 LC-MS analysis

The ribitol-methanol fruit extract was analysed using a Waters Acquity UPLC system coupled to a Q-Exactive Orbitrap mass detector according to a previously published protocol [3]. The UPLC system included a HSS T3 C18 reversed phase column (100 × 2.1 mm i.d., 1.8-µm particle size; Waters) that was set to a temperature of 40°C. The mobile phases consisted of 0.1% formic acid in water (Solvent A) and 0.1% formic acid in acetonitrile (Solvent B). The flow rate of the mobile phase was 400 µL min^−1^, and 3μl of sample was loaded per injection. The UPLC was connected to an Exactive Orbitrap (Thermo Fisher Scientific) via a heated electrospray source (Thermo Fisher Scientific). The spectra were recorded using full scan mode of negative ion detection, covering a mass range from m/z 100 to 1500. The resolution was set to 25,000, and the maximum scan time was set to 250 ms. The sheath gas was set to a value of 60, while the auxiliary gas was set to 35. The transfer capillary temperature was set to 150°C, while the heater temperature was adjusted to 300°C. The spray voltage was fixed at 3 kV, with a capillary voltage and a skimmer voltage of 25 and 15 V, respectively. MS spectra were recorded from minute 0 to 19 of the UPLC gradient. Molecular masses, retention time, and associated peak intensities were extracted from the raw files using RefinerMS (version 5.3; GeneData), and Xcalibur software (Thermo Fisher Scientific)[4].

### 2.4 Metabolite identification

Metabolite identification and annotation were performed using standard compound analysis, literature, and tomato metabolomics databases [4], [5], [6], [7]. Values were obtained as relative to the internal standards, ribitol and isovitexin for GC-MS and UPLC-MS, respectively.

## Declaration of Competing Interest

The authors declare that they have no known competing financial interests or personal relationships which have or could be perceived to have influenced the work reported in this article.

## Appendix Supplementary materials

Image, application 1
